# Relationship between HIV Stigma and Self-Isolation among People Living with HIV in Tennessee

**DOI:** 10.1371/journal.pone.0069564

**Published:** 2013-08-08

**Authors:** Carolyn M. Audet, Catherine C. McGowan, Kenneth A. Wallston, Aaron M. Kipp

**Affiliations:** 1 Institute for Global Health, Vanderbilt University and Department of Preventive Medicine, Vanderbilt University School of Medicine, Nashville, Tennessee, United States of America; 2 Division of Infectious Diseases, Department of Medicine, Vanderbilt University School of Medicine, Nashville, Tennessee, United States of America; 3 Vanderbilt University School of Nursing, Nashville, Tennessee, United States of America; 4 Vanderbilt Epidemiology Center, the Institute for Medicine and Public Health, Vanderbilt University, Nashville, Tennessee, United States of America; Tulane University, United States of America

## Abstract

**Introduction:**

HIV stigma is a contributing factor to poor patient outcomes. Although HIV stigma has been documented, its impact on patient well-being in the southern US is not well understood.

**Methods:**

Thirty-two adults participated in cognitive interviews after completing the Berger HIV or the Van Rie stigma scale. Participant responses were probed to ensure the scales accurately measured stigma and to assess the impact stigma had on behavior.

**Results:**

Three main themes emerged regarding HIV stigma: (1) negative attitudes, fear of contagion, and misperceptions about transmission; (2) acts of discrimination by families, friends, health care providers, and within the workplace; and (3) participants’ use of self-isolation as a coping mechanism. Overwhelming reluctance to disclose a person’s HIV status made identifying enacted stigma with a quantitative scale difficult.

**Discussion:**

Fear of discrimination resulted in participants isolating themselves from friends or experiences to avoid disclosure. Participant unwillingness to disclose their HIV status to friends and family could lead to an underestimation of enacted HIV stigma in quantitative scales.

## Introduction

Stigma occurs when a person is identified as “tainted” or “less desirable” and results in the individual being devalued in the eyes of society [1]. A diagnosis of HIV in the United States (US) has been associated with social stigma since the beginning of the epidemic, in part given the historical association of the disease with commonly stigmatized groups such as men who have sex with men (MSM) and injection drug users [2]. For people living with HIV (PLHIV), HIV stigma has traditionally comprised three domains: perceived, experienced/enacted, and internalized [3,4]. Perceived stigma refers to the belief that a person will be discriminated or judged negatively if their status is revealed. Experienced/enacted stigma refers to the actual events of discrimination experienced by PLHIV. Internalized stigma refers to the shame and negative self-image felt by those diagnosed with HIV infection. Perceived and experienced stigma may negatively affect HIV testing, retention in care and adherence through fear that being seen at an HIV clinic, missing work to attend appointments, or being observed taking medication will inadvertently disclose one’s HIV status [5–9]. Experienced, perceived and internalized stigma may lead to depression, low self-esteem, isolation and feelings of hopelessness or loss of control, which may in turn result in lost motivation to remain in care and to be adherent to treatment [10,11]. Studies of HIV stigma have focused on three levels: (1) the micro level, including individual psychological context (the stigmatizer and the stigmatized) and the disease characteristics (concealability, contagion risk, culpability); (2) the macro level, including media portrayal of HIV or groups of people associated with the disease; and (3) the meso level, including social networks and current healthcare practices [12].

Stigma surrounding HIV has been hypothesized to be a contributing factor to poor patient outcomes including treatment adherence and retention in care in the US [7–9,13–16]. The perception that HIV infection is associated with social deviance and immorality may result in greater stigma towards PLHIV in the south, given higher levels of religiosity and conservatism in that region [17,18]. Evidence suggests that lower levels of HIV knowledge, common in the southern US, also contribute to increased HIV stigma [2,19]. Nearly half of newly reported HIV infections are geographically located in the southern US, although this region accounts for only 37% of the US population [20]. Surveillance data also identify regional disparities in HIV outcomes, with 9 of the 10 highest case–fatality rates located in southern states [21,22] Although HIV stigma has been associated with a negative impact on patient health outcomes and quality of life globally [3,8,10,23–34], its impact on patient well-being in the southern US is poorly understood [35–38]. Previous stigma studies focusing on rural African American women [36] and barriers to sexually transmitted infection testing among men in the south [35] have documented high levels of perceived, experienced, and internalized stigma among participants. Improved understanding of the impact of HIV stigma in a diverse population of PLHIV in the south is an important first step towards developing tools to improve retention, adherence, and quality of life.

In this paper we report on 32 HIV-positive participants in middle Tennessee who completed in-depth, qualitative interviews regarding their experience with, and perceptions of, HIV stigma. The goals of the qualitative interviews were threefold: (1) to ensure the questions contained in two stigma scales [39,40] were accurately understood in this population; (2) to identify sources of HIV stigma and acts of discrimination faced by PLHIV; and (3) to assess the impact stigma has on the lives, self-image, and behavior of participants. This qualitative research was part of a larger study which sought to compare and revalidate two existing HIV stigma scales [39,40] among a larger sample of PLHIV in middle Tennessee, a region representative of the epidemic in the south [21]. The results of the quantitative study are discussed elsewhere.

## Methods

### Study Location

This study was conducted from August to October 2011 at the Vanderbilt Comprehensive Care Clinic (VCCC) in Nashville, Tennessee. The VCCC provides medical, psychiatric, and nutritional care to an estimated 80% of PLHIV in middle Tennessee. In 2010, more than 2,800 PLHIV were in active follow-up at the clinic.

### Participant Eligibility and Recruitment

Adult (≥ 18 years of age) PLHIV with at least one prior medical provider visit at the clinic were eligible to participate if they were able to communicate in English and capable of providing informed consent. Pregnant women were not included in the study as their care is intensive and highly structured. Participation in this study was considered to be overly burdensome to these women given the large amount of time already spent at the clinic. A convenience sample of patients attending the clinic was recruited and enrolled by approaching eligible patients while waiting in the exam room to be seen by the provider. Patients who visited a provider Monday to Thursday were recruited for the quantitative study (N=269). The quantitative methods and results of this comparative analysis are presented and discussed in detail elsewhere [Kipp et al. unpublished]. Those who attended clinic on Friday were recruited for the qualitative interviews (N=32). Patients who were interested and willing to participate met with study personnel in a private interview room following their clinic appointment to complete the interviewer-administered questionnaire. Participants were compensated for their time with a $20 gift card to a retail store upon completion.

Written informed consent was obtained before the interview began. Participants were first asked basic demographic questions, including age, gender, highest education level completed, and disclosure of their HIV-positive status. They then completed either the Berger HIV Stigma Scale [39] or the Van Rie HIV/AIDS-related Stigma Scale [40] which were alternated between consecutive participants to ensure that the appropriateness of the questions in each scale was probed equally. Upon completion of the stigma scale, stigma items and/or participant responses to items were probed through cognitive interviewing using a semi-structured interview guide that was amended throughout the study to adapt to new findings. Participants were asked to give an example of their own experiences or emotions elicited by each stigma scale question, whether each question was relevant to their situation and experiences, and whether their experiences with stigma had changed since their diagnosis. Interviews continued until participants expressed no new interpretations of the scale questions and no new themes related to stigma experiences emerged from the interviews. The interviews lasted between 30 and 90 minutes and were audiotaped.

Transcribed interviews were read by two of the authors (CMA and AMK). From an initial read of six transcripts we identified categories and themes inductively [41] which were applied to the analysis of the remaining 26 interviews. Using thematic analysis, we categorized text segments by themes (as identified in the first six interviews) and identified additional themes inductively as they were identified. Each interview was read a minimum of four times to ensure accurate categories were assigned. Sections of the text were marked and linked with similar themes found in other interviews using MAX QDA 2007 software (www.maxqda.com). The sections of text were analyzed in the context of the interview to minimize misunderstanding or misinterpretation [42]. Inter-rater reliability scores were not developed as the interview questions alternated depending on which scale was completed and according to the participants’ responses to the scale items; however AMK and CMA discussed the interpretation of the data regularly.

#### Ethics statement

This research was reviewed and approved by the Vanderbilt Institutional Review Board (IRB# 110829) and all participants provided signed informed consent.

## Results

We approached 78 patients to complete the in-depth, qualitative interview: 20 (25.6%) refused, 26 (33.3%) initially expressed interest but did not return, and 32 (41.0%) completed the in-depth interview. Patient time constraints appeared to be the major reason for refusal to participate. Of those who participated, 26 (81%) were male, the median age was 44 years (inter-quartile range: 37.5 to 52), 19 (59%) were white/Caucasian, 11 (34%) were African American, and the remaining 2 (6%) were self-reported mixed race or other. Seventy-two percent were high school graduates. Thirty (94%) participants were on antiretroviral medication (ART) at the time of the interview, and median time since HIV diagnosis was 10 years (IQR: 5 to 17.5). The 32 patients who participated in qualitative interviews were similar to those completing only the quantitative portion (n=269) of the study (all p-values >0.24), with two notable exceptions: qualitative participants were more often high school graduates (72% vs. 51%; p=0.02) and had a different distribution for likely route of HIV infection (heterosexual sex: 19% vs. 28%; unknown route: 28% vs. 11%; p=0.05). HIV stigma scores also did not differ between the qualitative and quantitative study participants (all p-values >0.25).

Participants described the moment they received their HIV diagnosis, how they (and their families) coped with that diagnosis, their perceptions and experiences of HIV stigma in the community, and their reactions to stigma. Three main themes emerged regarding HIV stigma and PLHIV in Middle Tennessee: (1) negative attitudes, fear of PLHIV, and misperceptions regarding HIV transmission existed; (2) acts of discrimination by families, friends, medical staff, and within the workplace were common; and (3) participants chose self-isolation as a coping mechanism to limit their exposure to negative reactions.

### Attitudes towards PLHIV in Middle TN

Most participants believed that attitudes towards people with HIV had improved in the last five years, but they also indicated that prejudice and negative attitudes towards PLHIV existed in their communities. Participants identified two major sources of stigma: (1) fear of contracting the virus due to misperception of transmission mechanisms; and (2) presumptions regarding the source of infection among PLHIV. Participants also internalized stigma, as some struggled with previous decisions they now deemed to be high-risk.

Misperceptions regarding the mechanisms and risks of HIV transmission were commonly experienced by participants. Family, friends, and even medical professionals had exposed their ignorance of HIV transmission by refusing to touch participants or share their dinner plates, and by bleaching items that they had touched. However, participants noted that those who were educated and/or younger were more likely to be non-judgmental of those living openly with HIV. One participant summed up people’s fears:

They really don’t know too much about it or how … you can get it or … things like that so they’ll probably feel like if I touch something they touch something they can get it from that.… they just don’t have the … knowledge of knowing … how a person can get it but it’s not contracted by me touching … or hugging or nothing. But some might think that you know (41 year old white, heterosexual, male).

Although lack of HIV knowledge was perceived to be the source of stigma among some participants, others suggested that negative attitudes are due to issues other than simple fear of contracting the virus. The route of infection was also perceived to be a source of stigma, particularly among religious people. One man explained:

You know it could be an ethical issue. I think a lot of people go into the, how did they get AIDS? Well they must have slammed some heroin or hooked up with [a] prostitute, or they’re gay, you know. You must be a sinner to get AIDS. Yeah, so I think that could have a play in it especially here in the Bible belt (51 year old white male).

Other participants had internalized HIV stigma. One women spoke of her HIV infection as a result of “being caught” doing risky things-- “if you steal, you get caught, you got to go to jail. So I did intravenous drugs, I had unprotected sex, so I got caught [and got HIV].” Many struggled with the perception that people with HIV are dirty or immoral, struggling to avoid adopting this view as their own:

I’m the cleanest person, I bathe, I brush my teeth, I do all that stuff, but somehow I ended up with it, this disease. It’s not a bad person’s disease, you ain’t got to be bad, you don’t have to be naughty, I mean [there are] reverends and preachers and all that [are] probably dealing with it (39 year old African American female).

### Discrimination Experienced by PLHIV

Participant responses revealed a delicate balance between finding social support and avoiding discrimination. Of the 15 participants who were interviewed regarding their responses to the Berger HIV Stigma Scale, seven stated that on at least one of the Enacted Stigma items (i.e., ‘I have lost friends due to my HIV status’) they disagreed with the statement. Although this was initially interpreted positively (suggesting low levels of discrimination), it was found that participants were disagreeing not because of positive experiences when others knew of their HIV status, but rather, they had not disclosed their status specifically for fear that the response would be one of discrimination. This likely led to an underestimation of discrimination by the Enacted Stigma subscale. For example, one woman explained why she disagreed:

I: Have you had any bad experiences telling anybody?

R: No, because, like I said, I haven’t told that many [people] and those that I have told are my friends and were nurses in the first place. So they understand. But like I say, I don’t go around just wearing a sign (*laughs*).

I: You don’t have a t-shirt [disclosing your status]?

R: …it’s just confusing [because] you never know how [a person is] going to react even if they have it [HIV] in their family (57 year old African American female).

#### Discrimination by Family Members

Overall, participants reported that family members who knew their status (68% had disclosed to a parent and 75% to another family member) were supportive of them after learning about their HIV diagnosis. Of those who chose not to disclose, they did so for one of two reasons: (1) they feared adding “unnecessary” stress to the family; or (2) they believed that the parent or sibling would not be supportive. Of those who chose to disclose, some reported stigma, particularly at the time of disclosure:

R: When I first told family most of them panicked, you know oh you know you can’t touch you, you can’t you know you got to take and spray everything in the house with Clorox.

I: So did you educate them? Has it changed?

R: Yeah I may, I said okay well let’s go down to the County help people [the health department] and talk to people down there and let them explain you know what they know and it took them time but they finally caught on to the idea okay you know so, I think most people are just misinformed or don’t know enough and it, it does scare them (60 year old white male).

#### Discrimination in the Community

Despite some negative reactions, few participants reported cutting ties with their family completely. PLHIV were willing to tolerate some negative behavior from family members in order to maintain the relationship. In contrast, friends and acquaintances who knew about a participant’s status tended to be either very supportive or walked away from the friendship altogether. Twenty-five participants (78%) reported telling a friend their diagnosis:

I have never been the same since then. I mean there are still some relationships that are there with a few friends who were long term friends who have kind of stuck it out with me. But there were several people I was very hurt that I was good friends with that they decided that they didn’t want to have anything more to do with me, you know, after they found out. And it was, it just kind of broke my heart as a person, you know, to think that they were judging me based upon the virus not on our previous relationship, you know (55 year old white male).

Participants who self-identified as MSM noted high levels of stigma from other MSM:

R: The people who seemed to be the worst about it are not so much the straight people. It’s other gay people.

I: Why?

R: They, I guess, they just, I think there’s such a fear in the gay community of the virus especially if you don’t have it that you want to keep as far as away from people who do as you can. You know, and there’s still this huge mentality on the part of gay people thinking that it won’t happen to me (54 year old white male).

#### Discrimination by health care providers

Discrimination in the health care setting was described by five participants (16%). Four descriptions of discrimination were experienced by the PLHIV themselves, but we also interviewed one PLHIV who had been employed as a nurse technician at a local hospital. She described the discriminatory practices enacted against PLHIV who were hospitalized at her facility. Manifestations of discrimination included overzealous preventive measures by hospital personnel, isolation of HIV-positive patients, and staff unwilling to perform routine services to patients with HIV.

Three of the descriptions of discriminatory practices involved dentists. Participants described being physically isolated and forced to attend dental clinics on “HIV-positive day” or being denied access to treatment. This discrimination led many participants to avoid dental care altogether or to withhold disclosure of their status to ensure treatment:

Here a couple of years ago I called a dentist and wanted to get in, and I felt it was my responsibility to let them know I was HIV positive. You know it’s a big responsibility. But anyway, I did tell him. He said he would consult with his nurses to see if they had a problem with it. And I get the call back and they refused to see me. So, it makes you struggle with wanting to tell people, even care providers, you know, as to whether or not you can get your care (51 year old white male).

#### Discrimination in the Workplace

Only seven participants (22%) indicated that they were currently employed. Of those who were still working, none had disclosed their status to their employers. Among those who were employed, some were receiving disability benefits, others had alternative means of support, and two were actively looking for employment. Five (16%) people noted that they had been fired or forced to quit once their HIV status was disclosed. Only one person spoke of challenging his employer’s behavior. The man who filed a complaint was successful but was affected by the stress of this event:

I worked for [company name removed] in Memphis and although people are not supposed to go on and look up people’s medical information all the medical files especially if you worked in the billing office or if you worked in corporate, were all public and anyone with the access could do that ... I started just keeping notes on things that they were [behaving] and eventually … I quit. And after I quit, I went to EEOC and … I won the suit against [company name removed]. But it was the awful-est time of my life. It really was. I mean for a person having to deal with HIV and then having to deal with an employer they didn’t want you there any longer and they did everything they could to get you out. It was really tough (55 year old white male).

### Isolation

Among those struggling with the stigma surrounding HIV, the majority (75%) reported self-isolation from friends, families, and community members. The fear of disclosure was so strong that many participants expressed an unwillingness to develop new friendships, were afraid to engage fully in their communities, or, in extreme cases, rarely left their home. Once a participant experienced rejection that was perceived to be based on their HIV status they were much less likely to disclose their status again. One man summarized his self-isolation. To go out and meet people is:

…too much effort, too much risk. Yeah, I, I don’t know so much about the younger set of people, but in my particular group you are afraid to tell anybody and so yeah you are kind of isolated, kind of set you in a little group by yourself (46 year old white male).

Although both women and men recognized this tendency to isolate themselves, fear of others learning their status often overshadowed their need for companionship.

I don’t have any friends. I got a girlfriend I talk to on the phone ... We just talk on the phone. Um, I stay in the house, I stay isolated, I’m, I’m very active in my church, I sing in the choir and I stay at church from 7 in the morning till 12 at noon on Sundays, so I’m very active in that, but other than that, I don’t go anywhere, I stay isolated and it’s not good (42 year old African American female).

You don’t allow them to get too close to your life, you know, or to your personal life. You don’t invite them into your home. You might join them for cocktails and you might even tell little white lies, but you just stay guarded (51 year old white male).

The fear of disclosure was always present to most participants.

Yeah. It’s just like, it’s like a curse. I mean because of everything I’ve been through with the way people have treated me and when people don’t treat you right or people disown your friendship and you feel like you beat yourself up over it. You know, you, I know it’s easier said than done, but people say oh, well, you shouldn’t let it affect you and you shouldn’t beat yourself up over it. But after, the longer you’re infected, the, I think the harder it is on a person … You know, you just, after a while, you start beating yourself up especially when you get sick … it’s hard to get beyond the point of saying, you know, you’re a good person. You didn’t deserve this. Somebody did this to you who is awful so don’t beat yourself up, but it’s not that easy to do (34 year old African American female).

The majority (62%) of our participants reported being in committed relationships which served as a great source of support. However for those who were single, the fear of passing the virus to others and potential rejection was most apparent when it came to trying to find a romantic partner.

When I first was diagnosed with HIV I didn’t date anybody for like a year because I just thought that it would be hard to date somebody without disclosing my status and then I was afraid that I would be rejected which [is what] happened (27 year old male; race: other).

Another man spoke of his rejection when opening up to a potential male partner.

I was there watching a film and this other guy that I knew, just not well, but he kind of wanted to get outta there and take off, you know? So I told him, he actually got up and walked away and never came back, never said a word, just got up and walked out the front door. So that was actually my first rejection if you want to call it that (44 year old white male).

The concern that the fear of disclosure would lead to non-adherence with medical care and visits was not supported by discussions (although participants were recruited among patients already engaged in care). Generally people were unconcerned, although a few had been “outed” after meeting someone they knew during clinical visits. One woman told about the time she ran into a caregiver she knew in the waiting room and had to decide whether to disclose or lie about her presence at the VCCC:

I ran into this guy. It was cool running into him, you know, but then his sister was wheel-chairing him in the building and so I was like, oh, my God. What am I going tell this woman? So I tried to play it off. Oh, I brought my dad up here, you know, cause she knows my dad [is HIV positive] (34 year old African American female).

Eventually she disclosed her status and was assured of confidentiality, but expressed her lingering concern that her status could be disclosed to mutual friends or acquaintances.

## Discussion

Stigma associated with HIV infection was experienced, perceived, and/or internalized by the majority (but not all) of our participants. PLHIV had experienced stigma in multiple ways: friends avoided personal contact; coworkers and family members separated food and utensils; and bosses orchestrated ways to avoid working with those living with HIV. Participants who had not experienced discrimination carefully choose to whom they disclosed their diagnosis in an effort to avoid discrimination.

Participants’ actual responses to items on each of the HIV stigma scales mirrored the qualitative findings with one exception. Enacted stigma was not accurately captured by the Berger HIV Stigma Scale, particularly among those who had disclosed their status to few people. Participants “strongly disagreed” to statements about enacted stigma, suggesting less stigma (as measured by discrimination). On the contrary, we discovered that many people had no experience being discriminated against because they were too afraid to disclose their status (e.g., perceived stigma). This practice likely resulted in the enacted stigma sub-scales to mis-represent the degree to which discrimination would take place, if people disclosed their status. To the extent that this practice occurs regularly when the Enacted Stigma subscale of the Berger HIV Stigma Scale is administered to larger numbers of participants, the results may not be interpretable as intended. While affirming the items suggests higher levels of stigma, disagreeing with the items may mean that discrimination truly does not occur, or alternatively that the occasion for discrimination has not occurred. Conclusions about the presence or absence of stigma may differ depending on which mechanism is at work. This also draws attention to the fine line between discrimination (a component of stigma) and non-disclosure (a consequence of anticipated/perceived stigma).

The experiences and perception that HIV stigma exists in their communities led many participants to avoid new friendships, jobs, or other experiences to ensure their status remained hidden. This should be a concern for health care practitioners. PLHIV who isolate themselves have higher rates of depression, non-disclosure to sexual partners [11], and poorer health outcomes [43]. Similar to studies among African Americans in the southern US, participants in our study who lived in rural areas expressed greater distress about disclosure, fearful that their status would be difficult to keep secret in a small community [8,36]. Many recognized this behavior as unhealthy, but were more concerned about exposing themselves or their families to stigma or discrimination. Disclosure often led to devastating consequences. Participants were fired from jobs, isolated from family members, and/or abandoned by friends. Few participants reported discrimination from medical providers; but as in other parts of the US, dentists were criticized for refusing to provide services [44].

Fear of stigma isolated people from the communities that once were comfortable and supportive. While participants noted the lessening of stigma among some segments of the population (younger, non-religious, and educated), HIV infection remained stigmatized. For example, HIV has historically been stigmatized in highly religious communities [45], due in part to the perception that people associated with HIV are living a “deviant” lifestyle (promiscuity, drug use, prostitution, and homosexuality). All of our participants spoke of this negative association and the difficulty it presented them. Dating was also challenging for most participants, particularly for young MSM. Similar to findings from other regions in the US, self-identified MSM commented that the MSM community in Nashville was not supportive of those open about their HIV status [46]. This was an important finding, contrary to our expectations, that HIV stigma remains high within the MSM community, where it is likely independent of any negative attitudes towards sexual orientation held by the general population. This stigma further led to reluctance of participants to disclose their HIV status to potential partners. Similar disclosure concerns have been noted in the southeast US among African American populations, specifically women, although our small sample of women may have limited our ability to identify this issue [47]. For PLHIV who are female, African American, or identify as MSM, there may be a compounding effect of stigma related to HIV, race, and/or sexual orientation, such that the effects of HIV stigma (e.g., fear of disclosure) are magnified. Such magnifying effects have been reported for those who belong to marginalized groups. Our study did not specifically probe these interactions but we believe this is an important area for further research.

Similar associations between stigma and illness have been made with people suffering from mental health problems [48], tuberculosis, and leprosy [49], and attempts to reduce stigma surrounding these diseases (including HIV) have been met with limited success. Participants expressed uncertainty about what could be done to reduce the stigma within their communities aside from further education: they believed that the conservative culture prevalent in the southern US would limit the effectiveness of any intervention to reduce stigma, although interventions have been successfully implemented in the south [19,45].

Limitations of this study include the high refusal rate and the recruitment of participants who were already receiving care from an HIV clinic. The high refusal rate could have biased the data, although the effect of this bias is unclear. It is possible that those with few experiences with stigma were less interested in participating in the study, thereby resulting in the over-reporting of stigma experiences. Alternatively, those who experienced high stigma may have been less willing to participate, thereby resulting in the under-reporting of stigma experiences. We only interviewed participants who spoke English fluently, which excluded many immigrants to the region who may have different perspectives on HIV stigma. Similarly, the median duration of time since HIV diagnosis was ten years in our study, and it is possible that the attitudes, perceptions and experiences of our participants do not adequately reflect those of PLHIV who were more recently diagnosed.

## Conclusion

Despite the improvement of HIV knowledge among the general population, HIV stigma continues to impact PLHIV in middle Tennessee. Participants felt that disclosing their status could result in losing friends, family support, or even their jobs. A common coping mechanism was self-isolation, which may have negative implications for mental and physical health. Measuring enacted/experienced HIV stigma proved difficult in this population as many participants did not disclose their status for fear of discrimination. As a result, further clarification may be needed regarding how best to measure discrimination, fear of discrimination, and anxiety about disclosure. As no efficacious stigma reduction programs have been scaled up effectively in this region, more work needs to be done to assist PLHIV with coping mechanisms other than self-isolation.
